# Evaluating HBsAg rapid test performance for different biological samples from low and high infection rate settings & populations

**DOI:** 10.1186/s12879-015-1249-5

**Published:** 2015-11-30

**Authors:** Helena Medina Cruz, Leticia de Paula Scalioni, Vanessa Salete de Paula, Elisangela Ferreira da Silva, Kycia Maria Rodrigues do Ó, Flavio Augusto Pádua Milagres, Marcelo Santos Cruz, Francisco Inácio Bastos, Priscila Pollo-Flores, Erotildes Leal, Ana Rita Coimbra Motta-Castro, José Henrique Pilotto, Lia Laura Lewis-Ximenez, Elisabeth Lampe, Livia Melo Villar

**Affiliations:** Laboratory of Viral Hepatitis, Oswaldo Cruz Institute, FIOCRUZ, Rio de Janeiro, Brazil; Laboratory of Technological Development of Virology, Oswaldo Cruz Institute, FIOCRUZ, Rio de Janeiro, Brazil; São Lucas Hospital, Petropolis, Rio de Janeiro, Brazil; Medicine Faculty, Federal University of Tocantins, Palmas, Brazil; Institute of Psychiatry, Federal University of Rio de Janeiro, Rio de Janeiro, Brazil; Institute of Communication and Scientific Information & Technology for Health, Oswaldo Cruz Foundation, Rio de Janeiro, Brazil; Antonio Pedro University Hospital, Federal Fluminense University, Rio de Janeiro, Brazil; Federal University of Rio de Janeiro, Campus Macaé, Rio de Janeiro, Brazil; Federal University of Mato Grosso do Sul and FIOCRUZ-MS, Campo Grande, MS Brazil; Laboratory of AIDS and Molecular Immunology, Oswaldo Cruz Institute, FIOCRUZ, Rio de Janeiro, Brazil; Present address: Viral Hepatitis Laboratory, Helio and Peggy Pereira Pavilion - Ground, Floor - Room B09, FIOCRUZ Av. Brazil, 4365 - Manguinhos, Rio de Janeiro, RJ 210360-040 Brazil

**Keywords:** Hepatitis B virus, Rapid tests, Performance of tests, Diagnostic procedures

## Abstract

**Background:**

Rapid tests (RTs) might have several advantages over standard laboratory procedures, increasing access to diagnosis, especially among vulnerable populations and/or those living in remote areas. The aim of this study was to evaluate the performance of RTs for the detection of hepatitis B virus surface antigen (HBsAg) in samples from different populations/settings.

**Methods:**

Three RTs for HBsAg detection (Vikia® HBsAg, HBsAg Teste Rápido®, and Imuno-Rápido HBsAg®) and different biological specimens (serum, whole blood, and saliva) were evaluated. Analyses comprised a reference panel and samples from field studies targeting suspected cases of hepatitis B virus (HBV) (G I), individuals living in deprived areas (G II), and highly vulnerable individuals (G III). Enzyme immunoassay (EIA) was defined as the gold standard in this study. Reproducibility, repeatability, and cross-reactivity with other infectious agents such as dengue, immunodeficiency (HIV), and hepatitis C (HCV) viruses and *T. pallidum* were determined.

**Results:**

For the reference panel, the sensitivity and specificity of all HBsAg RTs were higher than 93.00 %. G I presented the highest kappa values for all rapid assays using sera samples. When using serum, the sensitivity values were higher than 93.40 for G I, 60.00 % for G II and 66.77 % for G III, and the specificity values were higher than 99.50 for GI, 97.20 for G II and 99.10 % for G III for all tests. For whole blood samples & the Vikia® HBsAg assay, the best performance was achieved for GIII (k = 79.75 %). For saliva samples, the Imuno-Rápido HBsAg® assay showed the highest concordance values with EIA for G I (40.68 %) and G II (32.20 %). The reproducibility and repeatability of all RTs for serum and saliva were excellent, and the concordance between HBsAg EIAs and RTs using samples reactive with other infectious agents varied from 70.10 % to 100.00 %.

**Conclusions:**

The overall performance of RTs for HBsAg in serum was high/moderately high for all groups, thereby promoting increased access to HBV diagnosis among vulnerable populations as well as samples from individuals in emergency settings or remote areas. Rapid tests for HBsAg using whole blood could be used in prevalence studies, though these assays should not be used for saliva samples.

**Electronic supplementary material:**

The online version of this article (doi:10.1186/s12879-015-1249-5) contains supplementary material, which is available to authorized users.

## Background

Exposure to hepatitis B virus (HBV) may result in acute and chronic infections. Two billion individuals are estimated to have had contact with the virus and 240 million to be chronic carriers of HBV. Every year, approximately 600 thousand people die due to late complications of HBV infection [1].

Standard HBV diagnosis consists of the use of enzyme immunoassays (EIAs) and electrochemiluminescence (ECLIA) with serum or plasma samples [2]. However, these assays have limitations that may compromise their routine use in low- and middle-income countries: they require trained personnel as well as the availability of all necessary infrastructures. As an alternative, rapid tests (RTs) may have several advantages over standard procedures because they are easy to perform and can provide conclusive results within a few minutes. Additionally, these tests may be performed on a case-by-case basis and do not require laboratory infrastructure. Moreover, only minimal training is required to perform RTs [3–5].

Rapid tests for detection of the surface antigen of the hepatitis B virus (HBsAg) utilize a lateral flow device. Different approaches can be used in lateral flow assays, but in general, the patient’s sample is poured over a membrane containing two areas: the first contains antibodies against HBsAg (anti-HBs) for detection; and the second, the control area, contains a set of reagents that represents the quality control of the conjugate [4].

HBsAg RTs have been used for HBV clinical diagnosis and in serosurveys in different settings and countries [4–7]. In Brazil, although rapid tests for human immunodeficiency virus (HIV) and hepatitis C virus (HCV) have been widely used, as recommended by the Brazilian Ministry of Health (BMoH) [8, 9], no standard algorithm or guideline is yet available for HBV RTs. Before the implementation of rapid testing for HBV diagnosis, key parameters such as their sensitivity, specificity, cross-reactivity, reproducibility, and repetitively should be thoroughly evaluated.

The determination of the accuracy of a rapid test compared to a “gold standard” diagnostic procedure, such as ELISA, is key to minimizing false positive or negative results, thus increasing access to accurate diagnosis in remote areas and/or emergency settings. The aim of this study was to evaluate the performance of three rapid tests for HBsAg detection using three different types of fluid from individuals in different populations/settings.

## Methods

### Study population

No comprehensive population-based serosurvey in Brazil on HBV has been implemented to date. Local/focal studies have highlighted infection rates in different segments of the general population, and different at-risk groups are deeply heterogeneous. A comprehensive panel of sera from different key groups was established by the authors. Although based on convenience samples, it intentionally targeted as many populations and settings as possible, from all over the country.

The reference panel comprised serum samples obtained from 393 individuals recruited between 2010 and 2012 at Fiocruz Viral Hepatitis Ambulatory (Oswaldo Cruz Institute, Rio de Janeiro, Brazil), a Brazilian Referral center for the diagnosis of viral hepatitis (types A, B, C, D and E). The inclusion criteria for this group were acute, chronic or suspected cases of hepatitis B infections, age of more than 18 years and signed informed consent. Samples from individuals under follow-up at the Fiocruz outpatient clinic were tested for HBsAg using two ELISA kits (HBsAg, Radim, Pomezia, Italy and ETI-MAK-4, Diasorin, Italy) and three rapid tests (Vikia® HBsAg, Biomérieux, France; HBsAg Teste Rápido®, Doles, Brazil, and Imuno-Rápido HBsAg®, Wama, Brazil).

A field study was composed of three groups (I-III), with each participant providing serum, whole blood and/or saliva. The serum samples were tested for HBsAg using one ELISA kit (ETI-MAK-4, Diasorin, Italy), and all biological samples were assayed for all RTs evaluated.

Group I (G I) comprised 371 individuals referred to Fiocruz Viral Hepatitis Ambulatory (Oswaldo Cruz Institute, Rio de Janeiro) from 2009 to 2013. Inclusion criteria were attendance at Fiocruz Viral Hepatitis Ambulatory, residing in underserved and impoverished areas in Rio de Janeiro City (capital of Rio de Janeiro State) and a suspected case of viral hepatitis infection. This group was considered the high-risk group.

Group II (G II) comprised 881 individuals living in three (of the five) Brazilian macro-regions (Southeast, North and Midwest) and belonging to the general population, among which HBV prevalence has been low in recent years. None of these individuals were recruited in viral hepatitis ambulatory care settings, and this group was considered to be a low risk for HBV. These samples were obtained from individuals living in Rio de Janeiro State (Petrópolis and Macaé cities), Tocantis State (Tocantinópolis city), and Mato Grosso do Sul State (the Pantanal region) in 2009–2013 and who agreed to be tested within the context of public campaigns aiming to increase viral hepatitis diagnosis and the prompt referral of infected patients for treatment and care. The individuals from Mato Grosso do Sul State lived in communities of the Pantanal region, up to 385 km far from Campo Grande City (Mato Grosso do Sul State), whereas other individuals lived up to 217 km far (by river transportation) from the city of Corumbá (Mato Grosso do Sul State). The individuals from Tocantins State lived in rural communities from Tocantinópolis, 30 km far away from the urban area of the city. Such individuals belong to socially isolated populations living in deprived, underserved communities.

The individuals from Rio de Janeiro State were employees from a private hospital located in Petrópolis city who belonged to the middle-class as well as individuals living in underprivileged communities of Macaé city. Petrópolis city is located in a mountain region, and Macaé city is situated in the northern region of Rio de Janeiro State.

Our aim in this study was to assess as many individuals as possible from remote areas and/or deprived communities, as well as a small subgroup of people from the middle-class stratum. This subgroup falls short of a population-based repository, but deliberate efforts were made to establish a pool as diverse as possible, focusing on underserved populations that could benefit the most from extended testing strategies.

Finally, Group III (G III) was composed of 251 vulnerable individuals, including 158 beauticians and 93 heavy users of crack cocaine from Rio de Janeiro State.

All study participants and/or their legal guardians consented and signed informed consent forms prior to enrollment. Ethical approval for the study was issued by the Oswaldo Cruz Foundation Ethics Committee. Laboratory results were promptly returned to the patients’ physicians.

### Sample collection

Whole blood and serum samples were collected by venipuncture using vacutainer tubes, with (BD Vacutainer® containing the anticoagulant EDTA) and without (BD SST II Advance®) additives, respectively. Saliva samples were collected using commercial collection devices (Salivette®; Sarstedt, Germany) and were mixed with 1 ml of transport buffer. Oral fluid samples were centrifuged (1,400 × g for 10 min) and stored at −20 °C until assayed, as detailed elsewhere [10]. For the reference panel, only serum samples were assayed, whereas serum, whole blood and saliva the field studies were analyzed.

### HBsAg and HBV detection

Serum samples from the reference panel were tested for HBsAg markers using two commercial EIAs (HBsAg, Radim, Pomezia, Italy and ETI-MAK-4, Diasorin, Italy) following the manufacturers’ instructions. Only samples with concordant results defined by both assays were included in the study. Serum samples from the field study were assayed for HBsAg detection using a commercial EIA (HBsAg, Radim, Pomezia, Italy). All HBsAg-reactive samples in the EIA were retested in duplicate. All serum samples were assayed for total antibodies directed against the total core antigen (anti-HBc total) as well as anti-HBs using EIAs (Diasorin, Italy). Serum samples were also assayed for anti-HBc IgM, HBV “e” antigen (HBeAg) and antibodies against HBeAg (anti-HBe) using commercial EIAs and ECLIAs (Diasorin, Italy) when sufficient sample volume was available.

### Rapid test evaluation

Three HBsAg rapid tests were evaluated: Vikia® HBsAg (Biomerieux, France), HBsAg Teste Rápido® (Doles, Brazil), and Imuno-Rápido HBsAg® (Wama, Brazil). All the RTs are approved by the Brazilian National Health Surveillance Agency (ANVISA), which is responsible for the regulation, control and supervision of products and services that involve risk to public health. Vikia® HBsAg has CE IVD approval but does not have FDA approval or WHO registration. Imuno-Rápido HBsAg® does not have FDA approval or WHO registration; such information is not available for HBsAg Teste Rápido®.

All three tests are qualitative tests based on immunochromatographic techniques for lateral association of monoclonal and polyclonal antibodies specific for HBsAg. The Vikia® HBsAg allows the detection of the main *ad* and *ay* subtypes in serum, plasma and whole blood by adding 75 μl of each sample to the test platform. Capillary blood by fingerstick can also be used in this test.

According to the manufacturers’ instructions, the analytical sensitivity of the RTs is less than or equal to 2 IU/ml for Vikia® HBsAg and from 10 IU/ml for HBsAg Teste Rápido® and Imuno-Rápido HBsAg®.

Readings were available within 15 min (though for negative samples, it was necessary to wait up to 30 min to confirm the result). The Imuno-rápido HBsAg® and HBsAg teste rápido® tests allow the detection of HBsAg in serum samples, and both assays use 100 microliters of sample. The results can be assessed within 20 min.

All procedures for rapid tests were performed according to the manufacturers’ recommendations, except for the saliva samples, for which twofold increases in sample volume (i.e., 75 μl → 150 μl and 100 μl → 200 μl) were adopted to increase the sensitivity of HBsAg detection. The manufacturers’ recommendations do not include the analysis of saliva samples. However, previous attempts made by our research group with regard to hepatitis C [11] were successful and motivated the current attempt. In the present study, we extended saliva analyses by incorporating HBsAg detection. Serum and saliva were assayed for all HBsAg RTs, and whole blood samples were evaluated using the Vikia® HBsAg test because it is the only test specifically designed for the latter.

### Reproducibility and repeatability

To evaluate the reproducibility and repeatability of HBsAg rapid tests, four samples (2 serum and 2 saliva samples) were tested in eleven replicates, each by two different operators, for two consecutive days. One HBsAg-reactive and another HBsAg-non-reactive serum sample by EIA were included. HBV-negative individuals donated saliva samples. These saliva samples were then diluted (1:1) with an HBV-reactive serum sample.

The HBsAg rapid testing procedures were similar to the procedures described above for serum and saliva. kappa statistics were used to cross-compare the results of rapid tests and EIA.

### Cross-reactivity studies

Serum samples reactive for other infectious agents were included in the analysis to assess the cross-reactivity of the HBsAg rapid tests. Twenty serum samples reactive for dengue virus (five for each of the co-circulating serotypes: DENV-1, DENV-2, DENV-3 and DENV-4), 69 HIV-reactive serum samples, 49 *Treponema pallidum*-reactive serum samples, and 137 HCV-reactive samples were included. HBsAg was assayed using commercial EIA (HBsAg One, RADIM) and HBsAg rapid tests (Vikia® HBsAg; HBsAg Teste Rápido®, and Imuno-Rápido HBsAg®).

### Data analysis

The data analysis comprised samples with well-defined serology. Indeterminate samples detected by EIA were excluded. Socio-demographic, epidemiological, clinical, EIA and rapid test results were entered into an Access® database. Statistical analyses were performed using SPSS 20.0 for Windows (SPSS Inc., USA). Parameters associated with test performance were evaluated using GraphPad InStat Programs, version 3.01 (GraphPad Software, San Diego, USA) and MedCalc, version 9.2.1.0 (MedCalc Software, Mari-akerke, Belgium).

The RT results were cross-compared with the EIA results, which was defined for the sake of this study as the gold standard. Analytic categories were defined as follows: true positive results (TP – positive in both tests), true negative results (TN – negative in both tests), false positive results (FP – positive in RT and negative in commercial EIA), false negative results (FN – negative in RT and positive in commercial EIA).

The clinical sensitivity (Cs), specificity (S), positive predictive value (PPV), and negative predictive value (NPV) for each rapid test were evaluated, and their respective 95 % confidence intervals (95 % CI) were calculated. Contingency tables and respective statistics were used to cross-compare findings from different testing procedures and populations/settings.

Concordance between the panel’s results and the results from rapid tests was assessed by kappa statistics [12]. P-values (two-tailed) <0.05 were considered statistically significant.

## Results

### HBsAg rapid test performance using reference panels

The reference panel was composed of 393 individuals, 103 of which were HBsAg reactive (sera), whereas 290 samples did not show HBsAg according to EIAs. The mean age (± standard deviation) of the patients was 40.32 years (±14.78), and most were female (61.24 %). HBsAg was detected in 101, 98 and 96 of the samples, with sensitivities of 98.06, 95.15 and 93.20 % by Vikia® HBsAg, Imuno-Rápido HBsAg® and HBsAg Teste Rápido®, respectively (Table 1).

**Table 1:** Accuracy metrics (point estimates and 95%CIs) of three rapid tests compared to results of HBsAg One® and ETI-MAK-4®, enzyme immunoassays

Manufacturer	TP	FN	TN	FP	Sensitivity	Specificity	PPV	NPV	K (CI%)
HBsAg non-reactive/HBsAg reactive (*n* = 393)									
Vikia HBsAg®	101	2	290	0	98.06 % (93.16–99.76)	100.00 % (98.74–100.00)	100.00 % (96.42–100.00)	99.32 % (97.54–99.92)	98.68 % (96.85–100.00)
Imuno-Rápido HBsAg®	98	5	287	3	95.15 % (89.03–98.40)	98.97 % (97.01–99.79)	97.03 (91.58–99.38)	98.29 % (96.05–99.44)	94.7 % (91.07–98.33)
HBsAg teste rápido®	96	7	287	3	93.20 % (86.51–97.22)	98.97 % (97.01–99.79)	96.97 % (91.39–99.37)	97.62 % (95.16–99.04)	93.34 % (89.26–97.42)

Legends: *TP* True positive, *FN* False negative, *TN* True negative, *FP* False positive, *PPV* Positive Predictive Value, *NPV* Negative Predictive Value, *k* Kappa statistics, *n* number of observations (biological samples), *CI* confidence interval

According to the three RT assays, HBsAg false negative serum samples had a low optical density/cut-off value ratio (OD/CO) by EIA when compared to HBsAg true positive samples (concise information about the analytical detection limits is available in Additional file 1: Web Appendix 1).

Most of the HBsAg-reactive samples also presented anti-HBc total, whereas anti-HBc and anti-HBs were not detected in most of the HBsAg-non-reactive samples, independent of the group studied (21.60 to 65.70 %). In addition, anti-HBc IgM, HBeAg, anti-HBe were most frequently detected in the reference panel and group I (the serological characteristics of HBV markers is available in Additional file 1: Web Appendix 2).

### HBsAg rapid test performance using field samples

Overall, 3,273 biological samples were collected in the different field studies, and 1,503 serum, 1,268 whole blood and 502 saliva samples were included in the analyses. The mean age of the individuals was 32.63 (±18:43) years, 34.89 (±12.95) years and 34.06 (±16:15) years for those donating serum, whole blood and saliva, respectively. Most individuals were women (51.93 % for serum, 55.19 % for whole blood, and 52.93 % for saliva).

The number of serum, whole blood and saliva samples from the different groups can be summarized as follows: G I - 371, 108 and 185 individuals; G II - 881, 767 and 160 individuals; G III - 251, 393 and 157 individuals, respectively. Overall (all groups and serum samples), Vikia HBsAg® presented the highest kappa value (96.08 %), followed by HBsAg teste rápido® (88.41 %) and Imuno-rápido HBsAg® (87.62 %). The specificities of all RTs were higher than 97 %, and Vikia HBsAg presented the highest sensitivity (Table 2).

**Table 2:** Accuracy metrics (point estimates and 95%CIs) of three rapid tests compared to results obtained by enzyme immunoassay ETI-MAK-4® in serum samples according to the characteristics of the study population

Profile/Manufacturer (n)	TP	FN	TN	FP	Sensitivity	Specificity	PPV	NPV	K
Group I									
Vikia HBsAg ®(371)	160	7	204	0	95.81 % (91.54–98.30)	100.00 % (98.21–100.00)	100.00 % (97.72–100.00)	96.68 % (93.30–98.65)	96.17 % (93.36–98,98)
Imuno-rápido HBsAg® (371)	157	10	203	1	94.01 % (89.27–97.09)	99.51 % (97.30–99.99)	99.37 % (96.53–99.98)	95.31 % (91.53–97.72)	93.98 % (90.48–97.48)
HBsAg Teste rápido® (371)	156	11	203	1	93.41 % (88.53–96.67)	99.51 % (97.30–99.99)	99.36 % (96.51–99.98)	94.86 % (90.99–97.41)	93.43 % (89.77–97.09)
Group II									
Vikia HBsAg ®(881)	3	2	875	1	60.00 % (14.67–94.73)	99.89 % (99.37–100.00)	75.00 % (19.42–99.37)	99.77 % (99.18–99.97)	66.50 % (28.65–100.00)
Imuno-rápido HBsAg® (881)	3	2	852	24	60.00 % (14.67–94.73)	97.26 % (95.94–98.24)	11.11 % (23.55–29.18)	99.77 % (99.16–99,97)	17.96 % (0.00–49.02)
HBsAg Teste rápido® (881)	3	2	857	19	60.00 % (14.67–94.73)	97.83 % (96.63–98.69)	13.64 % (29.04–34.94)	99.77 % (99,16–99.97)	21.50 % (0.00–54.67)
Group III									
Vikia HBsAg ®(251)	4	2	245	0	66.67 % (22.27–95.67)	100.00 % (98.51–100.00)	100.00 % (39.76–100.00)	99.19 % (97.11–99.90)	79.61 % (51.46–100.00)
Imuno-rápido HBsAg® (251)	4	2	244	1	66.67 % (22.27–95.67)	99.59 % (97.75–99.99)	80.00 % (28.35–99.49)	99.19 % (97.11–99.90)	72.12 % (40.76–100.00)
HBsAg Teste rápido® (251)	4	2	243	2	66.67 % (22.27–95.67)	99.18 % (97.08–99.90)	66.67 % (22.27–95.67)	99.19 % (97.11–99.90)	65.85 % (32.65–99.05)
Overall									
Vikia HBsAg ® (1503)	167	11	1324	1	93.82 % (89.21–96.87)	99.92 % (99.58–100.00)	99.40 % (96.73–99.98)	99.18 % (98.53–99.59)	96.08 % (93.87–98.29)
Imuno-rápido HBsAg® (1503)	164	14	1299	26	92.13 % (87.14–95.63)	98.04 % (97.14–98.71)	86.32 % (80.57–90.86)	98.93 % (98.21–99.42)	87.62 % (83.83–91.41)
HBsAg Teste rápido® (1503)	163	15	1303	22	91.57 % (86.50–95.21)	98.34 % (97.49–98.96)	88.11 % (82.57–92.39)	98.86 % (98.13–99.36)	88.41 % (84.72–92.1)

Legends: *TP* True positive, *FN* False negative, *TN* True negative, *FP* False positive, *PPV* Positive Predictive Value, *NPV* Negative Predictive Value, *k* kappa statistics, *n* number of samples, *CI* confidence interval

For serum samples from G I, the sensitivity was 93.41, 94.01, and 95.81 % and specificity 99.51, 99.51, and 100.00 % using HBsAg teste rápido®, Imuno-rápido HBsAg® and Vikia HBsAg®, respectively. For samples from G II, sensitivity was 60.00 % for all tests, and specificity was 97.83, 97.26, and 99.89 % for HBsAg teste rápido®, Imuno-rápido HBsAg® and Vikia HBsAg®, respectively. With respect to G III, sensitivity was 66.77 % for all tests, and specificity was 99.18 , 99.59, and 100.00 % for HBsAg teste rápido®, Imuno-rápido HBsAg® and Vikia HBsAg®, respectively.

As observed in the reference panel, HBsAg false negative samples, as defined by RTs, presented low values of OD/CO by EIA compared to true HBsAg-positive samples (Additional file 1 available in Web Appendix 1).

The Vikia HBsAg® test performed using whole blood samples presented the highest kappa value for G III (79.75 %), followed by G I (72.7 %). Concordance was not determined among the G II samples because the test could not detect true positives. Immuno-Rápido HBsAg® demonstrated the highest kappa value with EIA for G I (40.68 %) saliva samples, followed by G III (32.20 %). For G III samples, none of the available RTs could detect true HBsAg-positive samples; thus, a concordance value could not be calculated (Table 3).

**Table 3:** Kappa statistics and positive and negative samples detected by rapid tests using saliva (3) and whole blood (1) samples compared to results obtained using respective serum samples by enzyme immunoassay ETI-MAK-4®^,^ according to the characteristics of the population under analysis

Profile/Manufactory – Biological specimen	TP	FN	TN	FP	K
Group I					
Vikia HBsAg® - SALIVA	6	76	101	2	5.92 % (0.00–21.8)
Imuno-Rápido HBsA® - SALIVA	33	49	101	2	40.68 % (26.82–54.54)
Teste rápido HBsAg®- SALIVA	2	80	101	2	0.55 % (0.00–16.61)
Vikia HBsAg® - WHOLE BLOOD	18	10	80	0	72.73 % (56.63–88.83)
Group II					
Vikia HBsAg® - SALIVA	0	2	153	5	^a^
Imuno-Rápido HBsA® - SALIVA	1	1	155	3	32.2 % (0.00– 97.8)
Teste rápido HBsAg® - SALIVA	0	2	158	0	^a^
Vikia HBsAg® - WHOLE BLOOD	0	3	763	1	^a^
Group III					
Vikia HBsAg® - SALIVA	0	4	153	0	^a^
Imuno-Rápido HBsA® - SALIVA	0	4	149	4	^a^
Teste rápido HBsAg® - SALIVA	0	4	150	3	^a^
Vikia HBsAg® - WHOLE BLOOD	4	2	387	0	79.75 % (51.76–100.00)
Overall					
Vikia HBsAg® - SALIVA	6	82	407	7	7.72 % (0,00–25,11)
Imuno-Rápido HBsA® - SALIVA	34	54	405	9	45.65 % (33.1–58.2)
Teste rápido HBsAg® - SALIVA	2	86	409	5	1.67 % (0.00–19.95)
Vikia HBsAg® - WHOLE BLOOD	22	15	1230	1	72.72 % (59.44–86.00)

Legend: *TP* True positive, *FN* False negative, *TN* True negative, *FP* False positive, *k* kappa statistics, *n* number of observations (biological samples), *CI* confidence interval

^a^not amenable to calculation

### Reproducibility and repeatability

Reproducibility and repeatability analyses comprised 528 tests (264 serum and 264 saliva samples). The HBsAg teste rápido®, Immuno-Rápido HBsAg® and Vikia HBsAg® assays exhibited a 100.00 % kappa value for the EIA results for all samples (i.e., serum and saliva samples HBsAg reactive/non-reactive).

### Cross-reactivity with other infectious agents

HIV-, dengue virus-, HCV- and *T. pallidum*-reactive samples were evaluated using HBsAg rapid tests and EIA. HBsAg-reactive results were found among eight HCV samples, nine *T. pallidum* samples, and 15 HIV samples using EIA. In contrast, no HBsAg-reactive sample was found among the DENV samples.

The kappa statistic for EIA and RTs for HBsAg detection varied from 76.60 % to 85.10 % for *T. pallidum*, 70.10 % to 81.10 % for HIV, 78.80 % to 86.70 % for HCV and 100 % for dengue for all manufacturers. False negative results were found for all manufacturers for *T. pallidum*, HIV and HCV, and false positive results were found in one HIV-reactive sample (using HBsAg teste rápido®) and two HCV-reactive samples (one by HBsAg teste rápido® and the other by Immuno-Rápido HBsAg®) (Table 4).

**Table 4:** Kappa statistics and positive and negative samples detected by HBsAg rapid tests among serum samples with reactive serology for different infections compared to results obtained by enzyme immunoassay ETI-MAK-4 ®

	TP (n)	FN (n)	FP (n)	TN (n)	K% (IC%)
Dengue (*n* = 20)					
Vikia HBsAg®	0	0	0	20	100.00 %
Imuno-Rápido HBsAg®	0	0	0	20	100.00 %
Teste rápido HBsAg®	0	0	0	20	100.00 %
*T. pallidum* (*n* = 49)					
Vikia HBsAg®	7	2	0	40	85.10 %
Imuno-Rápido HBsAg®	6	3	0	40	76.60 %
Teste rápido HBsAg®	7	2	0	40	85.10 %
HIV (*n* = 69)					
Vikia HBsAg®	11	4	0	54	81.10 %
Imuno-Rápido HBsAg®	9	6	0	54	70.10 %
Teste rápido HBsAg®	11	4	1	53	77.00 %
HCV (*n* = 137)					
Vikia HBsAg®	6	2	0	129	85.00 %
Imuno-Rápido HBsAg®	6	2	1	128	78.80 %
Teste rápido HBsAg®	7	1	1	128	86.70 %

Legend: *TP* True positive, *FN* False negative, *TN* True negative, *FP* False positive, *k* kappa statistics, *n* number of observations (biological samples), *CI* confidence interval

## Discussion

The present study demonstrates the usefulness of HBsAg rapid tests in both the lab and the field. All rapid tests detected HBsAg among serum samples from a reference panel, with high sensitivity and specificity within contexts of high background prevalence. However, sensitivity was found to be poor when the background prevalence was low. In the field study, HBsAg rapid tests demonstrated a higher than 96.00 % specificity among serum samples, regardless of the manufacturer and the group under study, demonstrating the ability of these assays to detect HBsAg true negative samples [5, 12–15). The performance of RTs could be influenced by the antigen concentration because false negative samples had low average values of OD/CO compared to true positive samples, in agreement with previous studies [4, 14].

In the present study, the presence of HBV markers, such as anti-HBc or anti-HBs, did not appear to influence the HBsAg RT results. However, it was not possible to evaluate all the samples for HBeAg, anti-HBe and/or anti-HBc IgM due to low sample volumes. Thus, it was not possible to evaluate the influence of these markers on the performance of HBsAg RT performance.

The Vikia HBsAg® test presented the best performance in both the laboratory and field for serum samples, and the highest concordance was observed among G I patients. Such optimal performance may be secondary to the high HBsAg prevalence in this group, as observed by Lien et al. [13] using RTs from other manufacturers. In Brazil, confirmed HBV cases from 1999 to 2011 (120.343) were most reported in the southeast region (36.30 %), where Rio de Janeiro is located (16).

Low HBsAg prevalence could influence the performance of rapid tests, as found for the G II and G III samples. In Brazil, the HBsAg prevalence varies from 0.63 % in the northern region to 0.31 % in both the southeastern and midwestern regions [16]. Previous studies conducted among beauticians and crack users showed a lower HBsAg prevalence of 0 % to 6.2 % in Rio de Janeiro [17, 18]. However, in the present study the prevalence in these groups was 2.3 %, corroborating the a priori assumption these groups are particularly vulnerable to HBV infection.

The best performance of the Vikia HBsAg® test in whole blood samples was observed in the G III samples, favoring the applicability of this rapid test to vulnerable individuals. Nonetheless, the kappa value was relatively low (72.73 %) compared to a previous study conducted in France (96.98 %) [4]. The observed difference may be secondary to different blood collection procedures: blood was obtained by venipuncture in tubes without anticoagulant in the previous study [4] yet by venipuncture in tubes with anticoagulant in the present study.

The best performance for saliva samples was achieved using the Immuno-Rápido HBsAg® assay in the G I and II samples. However, a high number of false negative results were observed for the tests from all manufacturers, regardless of the group under study, which is most likely secondary to the low concentration of HBsAg in saliva samples [19]. In addition, HBsAg rapid tests were not originally developed for saliva samples, which may also explain the low concordance with EIA results.

Accordingly, our findings must be viewed as a warning against the use of these procedures for HBsAg detection in saliva samples. The previous promising findings respecting hepatitis C testing were, unfortunately, not observed for HBsAg.

In the present study, the volume of saliva sample was increased in all rapid tests to improve the sensitivity of the assay, but this procedure could not be translated into concrete benefits. Additional modifications, such as a longer incubation period, may be pursued by future studies.

The HBsAg rapid tests demonstrated excellent repeatability and reproducibility in serum and artificially contaminated saliva samples, demonstrating the good performance of these assays under laboratory conditions.

Although two meta-analysis studies showed high pooled accuracy for RTs for HBsAg [20, 21], Khuroo et al. [21] also observed wide variation in sensitivity among individual tests (43.5 % to 99.8 %), which could be due to the design of the studies or population characteristics because the performance of RTs are better in developed than in developing countries. In the present study, a wide variation of sensitivity of RTs for HBsAg was observed according to the group studied (60.00 % to 95.81 %), demonstrating the importance of evaluating RTs among specific populations before implementation at a large scale.

Regarding cross-reactivity, the best results using serum samples were found among reactive dengue samples, most likely due to the absence of HBsAg-reactive samples and/or the low number of dengue samples under analysis (i.e., due to beta error). Regardless, for *T. pallidum-*, HIV- and HCV-reactive samples, false negative and positive HBsAg results were observed for all RTs. However, the poorest performance was observed among HIV-reactive samples. This is of concern because under real life conditions, a non-negligible fraction of patients may be co-infected by the two viruses, which may yield confusing results and inconclusive clinical interpretations.

The Vikia HBsAg® and HBsAg teste rápido® tests showed better kappa values for HIV- and HCV-reactive samples, respectively, and both Vikia HBsAg® and HBsAg teste rápido® showed better kappa values for *T. pallidum*-reactive samples. These results suggest that both assays may have good performance with samples reactive for other infections. Other HBsAg RTs presented good performance among HIV-reactive samples [6, 7]. In addition, a meta-analysis demonstrated that co-infections (for example HIV, HCV, tuberculosis) did not influence the diagnostic accuracy of HBsAg RTs, which could be helpful for the adoption of these assays in endemic areas where these co-infections are highly prevalent [21].

This study presents some limitations, such as the absence of HBsAg neutralization, HBV DNA testing or HBsAg concentration due to low sample volume. However, reactive HBsAg samples were retested in duplicate to confirm the HBsAg results.

## Conclusion

In conclusion, the present study showed moderate to high concordance of HBsAg rapid tests using serum samples from different populations/settings. These findings could be useful for HBV diagnosis among individuals who are highly vulnerable to HBV infection as well as those recruited from emergency settings or remote areas. In addition, saliva samples should not be used for HBsAg detection with the assays evaluated in the present study.

## Additional file

Additional file 1:
**Appendix 1.** Mean values of OD/CO from EIE among false negatives and true positive rapid tests for HBsAg detection in three rapid tests according to the characteristics of the study population. **Appendix 2.** HBV markers (anti-HBc, anti-HBs, anti-HBc IgM, HBeAg, anti-HBe) detected in serum samples using enzyme immunoassay according to the population studied. (DOC 63 kb)

## Abbreviations

Anti-HBc IgM: Antibodies directed against the core antigen IgM
Anti-HBc total: Antibodies directed against the core antigen
Anti-HBe: Antibodies against HBeAg
ANVISA: National Health Surveillance Agency
Anti-HBs: Antibodies directed against hepatitis B surface antigen
BMoH: Brazilian Ministry of Health
CI: Confidence interval
CO: Cut-off value
Cs: Clinical sensitivity
ECLIA: Electrochemiluminescence
EIA: Enzyme immunoassay
FDA: Food and drug administration
FN: False negative result (negative in RT and positive in commercial EIA)
FP: False positive result (positive in RT and negative in commercial EIA)
HBeAg: HBV “e” antigen
HBsAg: Surface antigen of the hepatitis B virus
HBV: Hepatitis B virus
HCV: Hepatitis C virus
HIV: Human immunodeficiency virus
NPV: Negative predictive value
OD: Optical density
PPV: Positive predictive value
S: Specificity
TN: True negative result (negative in both RT and EIA)
TP: True positive result (positive in both RT and EIA)
