# Supplementary material for: Evaluating HBsAg rapid test performance for different biological samples from low and high infection rate settings & populations
Source: BMC Infect Dis. 2015 Nov 30;15:548. doi: 10.1186/s12879-015-1249-5 (PMC4665901; doi:10.1186/s12879-015-1249-5)
Supplement: Additional file 1: — Appendix 1. Mean values of OD/CO from EIE among false negatives and true positive rapid tests for HBsAg detection in three rapid tests according to the characteristics of the study population. Appendix 2. HBV markers (anti-HBc, anti-HBs, anti-HBc IgM, HBeAg, anti-HBe) detected in serum samples using enzyme immunoassay according to the population studied. (DOC 63 kb) [file 12879_2015_1249_MOESM1_ESM.doc]

Appendix 1

Mean values of OD/CO from EIE among false negatives and true positive rapid tests for HBsAg detection in three rapid tests according to the characteristics of the study population

|  |  | Enzyme immunoassay | Vikia HBsAg®  OD/CO (mean ± SD) | | Imuno-rápido HBsAg  OD/CO (mean ± SD) | | HBsAg teste rápido®  OD/CO medium ± SD | |
| --- | --- | --- | --- | --- | --- | --- | --- | --- |
|  |  |  | HBsAg  FN | HBsAg  TP | HBsAg  FN | HBsAg  TP | HBsAg  FN | HBsAg  TP |
| *Reference Panels* | | HBsAg one (Radim) | 2.60±1.63 | 64.01±10.14 | 21.59±22.23 | 64.92±8.35 | 36.49±30.77 | 64.73±8.00 |
|  |  | ETI-MAK-4 (Diasorin) | 4.64 ± 1.29 | 75.07±9.90 | 39.03±32.48 | 75.47±9.63 | 53.00±33.25 | 75.21±10.09 |
| *Field Study* | High prevalence | ETI-MAK-4 (Diasorin) | 24.77±25.28 | 70.81±10.69 | 35.50±27.56 | 71.00±10.69 | 43.30± 32.09 | 70.68± 10.93 |
|  | Low Prevalence | ETI-MAK-4 (Diasorin) | 2.82±1.19 | 79.00±2.94 | 2.82±1.19 | 79.00±2.94 | 2.82±1.19 | 2.12±0.26 |
|  | High vulnerability | ETI-MAK-4 (Diasorin) | 2.12±0.26 | 67.10±6.55 | 2.12±0.26 | 67.10±6.55 | 79.00±2.94 | 67.10±6.55 |
|  | Overall | ETI-MAK-4 (Diasorin) | 16.66±22.59 | 70.82±10.20 | 26.06± 27.67 | 71.71± 10.08 | 32.41±32.94 | 70.70±10.35 |

**Legends: TP = True positive; FN= False negative; TN = True negative**; **FP = False positive; OD= Optical Density; CO= Cut-off; SD= Standard Deviation**

Appendix 2

HBV markers (anti-HBc, anti-HBs, anti-HBc IgM, HBeAg, anti-HBe) detected in serum samples using enzyme immunoassay according to the population studied.

| Markers | Reference Panel (n=393)  (frequency/%) | Field study | | |
| --- | --- | --- | --- | --- |
|  |  | High prevalence (n=371)  (frequency/%) | Low Prevalence (881)  (frequency/%) | High vulnerability (n=251)  (frequency/%) |
| HBsAg + |  |  |  |  |
| Anti-HBc total - / Anti-HBs - | 4 / 1.0 | 5 / 1.3 | 2 / 0.2 | 3 / 1.2 |
| Anti-HBc total + / Anti-HBs - | 99 / 25.2 | 162 / 43.7 | 3 / 0.3 | 3 / 1.2 |
| HBsAg - |  |  |  |  |
| Anti-HBc total + /Anti-HBs - | 17 / 4.3 | 38 / 10.2 | 8 / 0.9 | 4 / 1.6 |
| Anti-HBc total + /Anti-HBs + | 12 / 3.1 | 33 / 8.9 | 92 / 10.4 | 15 / 6.0 |
| Anti-HBc total -/Anti-HBs + | 70 / 17.8 | 53 / 14.3 | 197 / 22.5 | 67 / 26.7 |
| Anti-HBc total - /Anti-HBs - | 191 / 48.6 | 80 / 21.6 | 579 / 65.7 | 159 / 63.3 |
| Other markers |  |  |  |  |
| Anti-HBe + | 58 / 14.75 | 111 / 29.9 | 64 / 7.3 | 17 / 6.8 |
| HBeAg + | 9 / 2.3 | 20 / 5.4 | 0 / 0.0 | 1 / 0.4 |
| Anti-HBc IgM + | 12 / 3.1 | 17 / 4.6 | 0 / 0.0 | 0 / 0.0 |

**Legend: n = number of observations (biological samples); + = positive; - = negative**
